# Recent Advances on Starch-Based Biomaterials: A Review

**DOI:** 10.1021/acspolymersau.5c00188

**Published:** 2026-02-19

**Authors:** Renan N. Araújo, Bruna S. Bitencourt, Samile B. de Aguiar, Pedro A. I. Sponchiado, Petrus N. Kirsten, Larissa Tessaro, Ana Paula Ramos, Pedro E. D. Augusto, Bianca C. Maniglia

**Affiliations:** † Department of Physical Chemistry, São Carlos Institute of Chemistry (IQSC), 42512 University of São Paulo (USP), São Carlos 13566-590, Brazil; ‡ Department of Agri-food Industry, Food and Nutrition (LAN), Luiz de Queiroz College of Agriculture (ESALQ), University of São Paulo (USP), Piracicaba 13418-900, Brazil; § Department of Chemistry, Faculty of Philosophy, Sciences and Letters of Ribeirão Preto (FFCLRP), University of São Paulo (USP), Ribeirão Preto 14040-901, Brazil; ∥ Department of Physics, Faculty of Philosophy, Sciences and Letters of Ribeirão Preto (FFCLRP), University of São Paulo (USP), Ribeirão Preto 14040-901, Brazil; ⊥ European Centre for Biotechnology and Bioeconomy (CEBB), Université Paris-Saclay, Pomacle 51110, France

**Keywords:** starch, modified starch, structure and physical properties, biomaterials, wound dressings, drug delivery systems, scaffolds, hybrid and smart biomaterials

## Abstract

Starch’s versatility inspires biomaterials for biomedical uses, customized through modifications, blending, or substituents. Diligent efforts have been dedicated to the development of starch-based biomaterials, leveraging the material’s inherent biocompatibility and biodegradability, while aligning with environmentally sustainable considerations. While promising, most studies lack in vivo data and scalability assessments. In many cases, the reported advances are restricted to in vitro evaluations with limited information on long-term performance, clinical translation, and large-scale manufacturing feasibility in both economic and operational terms. This review furnishes an up-to-date synthesis of information available in the literature concerning recent breakthroughs in utilizing starch as a biomaterial, primarily focusing on advancements in areas such as wound dressings, drug delivery systems, the creation of scaffolds for regenerative medicine, and applications in tissue engineering. Advances have been made, with biomaterials presenting adequate biodegradability rates, active functions, good biocompatibility, and mechanical properties. However, it is noted that most research has not yet reached in vivo evaluations and lacks notions of large-scale production, in both economic and operational terms.

## Graphical abstract

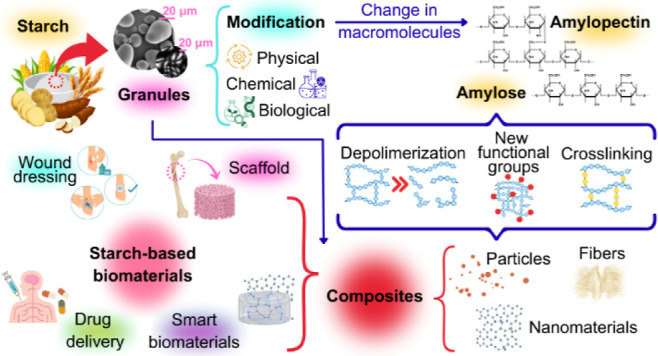

## 1 Introduction

Biomaterials are natural or synthetic materials designed to interact with biological systems to augment or replace tissue functions.^1^ Synthetic polymers are widely used due to their ease of processing, thermal stability, and superior mechanical properties; however, residual monomers or impurities may compromise biocompatibility and inhibit cell growth, which has increased interest in biopolymers derived from renewable sources with intrinsic biocompatibility and biodegradability.^2^

Thus, there is an emerging investigation of biopolymers for the preparation of materials for biomedical applications. Biopolymers derived from animal (e.g., gelatin, chitosan, and collagen) and plant (e.g., starch, cellulose, and pectin) sources have been extensively explored for biomedical applications.^3–5^ Each of these biopolymers presents distinct advantages and limitations in terms of availability, processability, mechanical performance, degradation behavior, and biological activity.

Among plant-derived biopolymers, starch stands out due to its abundance, low cost, and versatility and can be used for a variety of applications in different industries. It is a biopolymer that is found mainly in seeds, roots, and tubers. This polysaccharide is stored as granules ranging from 2 to 100 μm in diameter.^6^ The unique properties of starch make it extremely versatile, being thus important for several areas, for example, as a texture modifier in foods,^7^ nanocarriers for nutraceutical delivery,^8^ sizing agent in textile industry,^11,12^ sustainable packaging,^13^ biodegradable plastics,^9,10^ and as inks for 3D printing of scaffolds^14^ and food,^15,16^ among other applications (Figure 1A).

**Figure 1:**
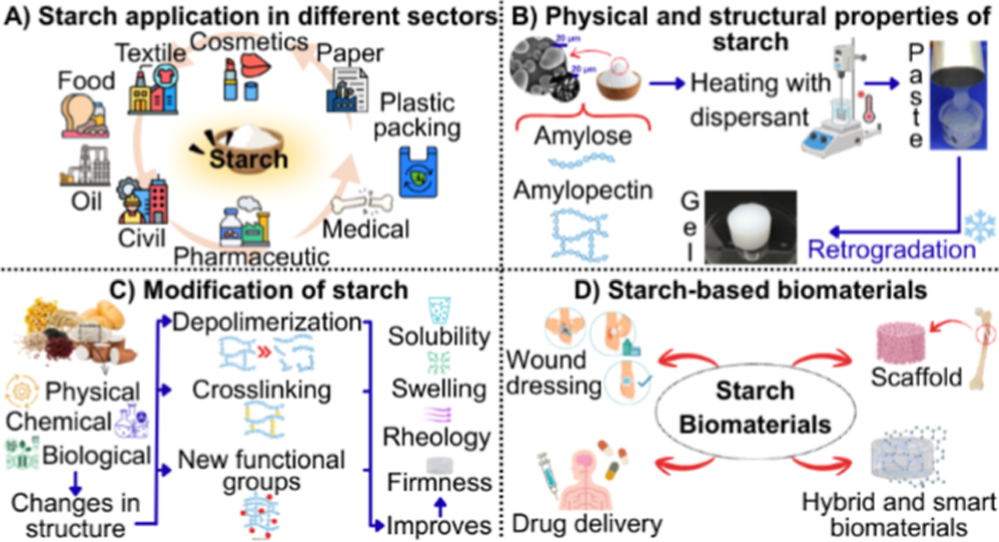
(A) Starch application in different sectors; (B) physical and structural properties of starch; (C) modification of starch; and (D) starch-based biomaterials.

Starch-based biomaterials have been investigated for various biomedical applications (Figure 1D) such as wound dressing;^17–19^ bone regeneration and replacement;^20–22^ drug delivery systems, adhesion, proliferation, differentiation, and regeneration of cells;^18,23^ and starch-based hybrid systems.^108,109^ Starch has been one interesting biopolymer for biomedical application mainly by presenting the appropriate criteria such as biocompatibility and the ability to regulate the degradation time, particularly advantageous for temporary implants such as bone scaffolds, where material resorption should match tissue regeneration rates.^24,25^ Native starch has processing issues, excessive swelling, high hydrophilicity, instability, and poor mechanics.^26^ Starch modification methods (physical, chemical, and biological) have been explored to improve the shortcomings of native starches and increase the utility of this biopolymer for a variety of applications (Figure 1C).

The production of starch-based biomaterials can involve different methods such as laser sintering, casting, freeze-drying, chemical cross-linking in molds, electro-spinning,^27^ and more modern methods such as additive manufacturing using 3D printing.^15,16^ In particular, starch-based inks are well suited for extrusion-based 3D printing, as their gel-like structure provides viscoelasticity, shear-thinning behavior, and sufficient shape fidelity during and after printing.^16^

This review discusses recent advances in wound dressings, drug delivery, scaffolds, and tissue engineering, highlighting gaps, such as the lack of in vivo testing.

## 2 Physicochemical and Structural Properties of Starch

### 2.1 Starch Structure: Molecules and Granules

From a chemical perspective, starch is a copolymer composed of two macromolecular complexes: amylose and amylopectin (Figure 1B). The botanical origin of starch affects the proportions, structure, size, distribution, and branching.^28^ Amylose is an essentially linear molecule composed almost entirely of d-glucopyranose linked by α-1,4 linkage, while amylopectin exhibits a highly branched structure, being larger than amylose, and composed mostly of d-glucopyranose linked by α-1,4 and interconnected by α-1,6 linkages, which form the branches.^28,29^

Differently from other biopolymers, starch molecules are not found free in vegetables but so organized in highly complex and semicrystalline granules. Starch granules are formed by a hilum in the center, which is believed to be the origin of the granule growth. The hilum is surrounded by amylose and amylopectin molecules arranged radially, forming a block structure with semicrystalline aggregates inside the granules. Amylose is more common in amorphous regions of starch granules, while amylopectin increases crystallinity through branch interactions^30,31^ (Figure 2).

**Figure 2:**
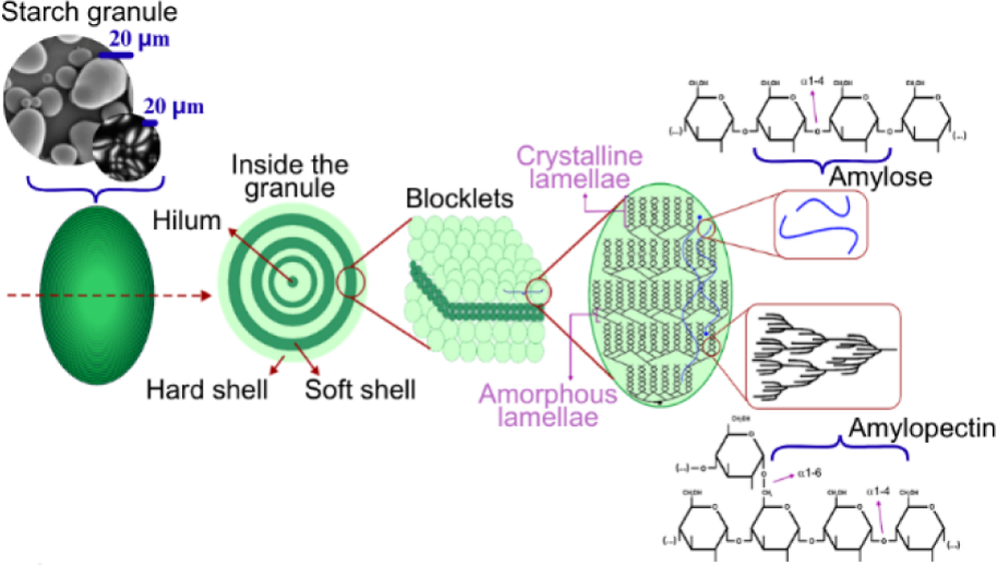
Starch granule structure.

The characteristics of starch granules make the properties of starches quite complex. Therefore, these properties need to be understood before designing new applications, as discussed in the next sections.

### 2.2 Gelatinization and Retrogradation

Starch is essentially insoluble in cold water or at room temperature. However, when starch is heated to gelatinization temperature in the presence of water, different mechanisms change the granule, resulting in a high interaction with water—which is referred to as starch gelatinization. This process promotes several irreversible changes in the starch granules, for example, the rupture of the semicrystalline structure, evidenced by the loss of birefringence, swelling/increase in the size of the granules, starch solubilization, and increase in consistency (viscosity).^29,32^ Heating weakens crystalline bonds, which allows water binding, granule swelling, and paste formation through amylose leaching.^33^ In addition, the leaching of amylose from the swollen granules plays an important role in increasing the paste consistency.^34^

After gelatinization, by cooling the paste, the starch molecules tend to reassociate into an ordered structure. This process is known as retrogradation and results in the formation of a gel with changes in consistency, opacity, and firmness. The reassociation of amylose molecules is much faster than amylopectin due to its molecular size, creating firmer gels; consequently, starches high in amylopectin yield softer, with a cohesive and elastic structure.^28,35,36^ High realignment poses a risk of syneresis (i.e., water being expelling from the gel).

Among the methods used to study starch gelatinization and retrogradation, the differential scanning calorimetry (DSC) and rapid vibration analysis (RVA) techniques are the most used for this purpose. DSC is used to measure thermal transitions of starch, providing information on gelatinization, melting, crystallization, and glass transition phenomena during heating and cooling.^38^ RVA, in turn, evaluates paste behavior under shear, allowing the evaluation of key viscosity-related parameters such as peak apparent viscosity (PAV), trough apparent viscosity (TAV), breakdown (BD), final apparent viscosity (FAV), setback (SB), and pasting temperature (PT).^39^

Surely, it is important to emphasize that gelatinization and retrogradation processes occur in different ways in different starch sources and are influenced by several factors such as amylose and amylopectin contents, whether modified or native, gelatinization temperature, starch, and water concentration. Thus, understanding the starch gelatinization and retrogradation processes is essential when developing starch-based biomaterials, hence making it possible to adjust the process conditions to obtain materials with tailored properties for specific applications.

### 2.3 Swelling and Solubility

When starch granules are heated in excess water, the crystalline structure of the granule ruptures, and hydrogen bonds are formed between starch components and water, resulting in increased granule swelling and solubility.^40,41^ The swelling power and solubility are affected by the amylose/amylopectin ratio and the characteristics of these molecules.^42^ In fact, the swelling power and solubility are different for each starch source, with potato starch standing out with the highest swelling capacity compared to other known sources.^43^

Overall, the swelling and solubility properties of starch granules make them valuable for many applications, particularly in the field of biomaterials and tissue engineering. For example, starch can be applied in the encapsulation of bioactive compounds and modulation of the controlled release of drugs, allowing the encapsulated compound to be digested differently.^44^ In addition, tissue-engineered scaffolds with adequate swelling properties allow control of the diffusion of bioactive compounds and the adhesion and migration of cells through the complex network structure.^45^ Furthermore, the capacity to absorb moisture and consequent swelling of starch may facilitate the enzymatic degradation of starch-based biomaterials and provide a convenient environment for the enzymatic activity of amylase. Therefore, modulating starch swelling and solubility opens many options for the development of versatile and functional biocompatible biomaterials.

### 2.4 Paste and Gel Properties: Rheology

The sequences of changes that starch granules undergo during gelatinization and retrogradation affect the rheological behavior of the system (suspension, paste, and gel). The rheological behavior of starch is complex and unique due to the granular structure and presence of amylose and amylopectin molecules; however, it is essential for determining the process conditions depending on the desired application.

The gelatinization process and paste properties are normally evaluated in starch using an amylograph, such as the RVA.^38,46,47^ This latter is easy to operate equipment and can measure the apparent viscosity variation throughout the gelatinization and beginning of retrogradation processes, providing useful parameters that describe the process. The test is divided into five steps (Figure 3). The literature provides useful parameters that describe this process.^37,47^

**Figure 3:**
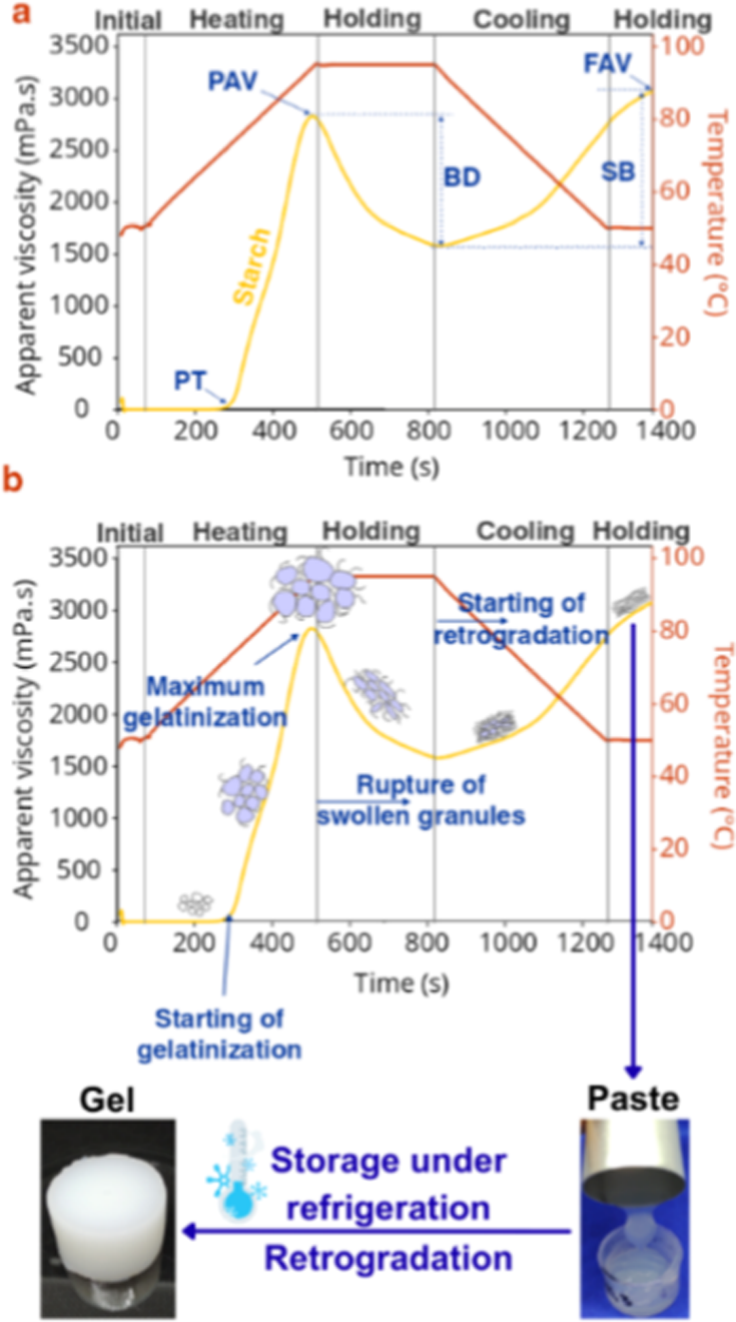
Pasting graph obtained by RVA for corn starch (as an example), where (a) main steps of the starch gelatinization and parameters obtained (PAV: Peak Apparent Viscosity; BD: Breakdown; FAV: Final Apparent Viscosity; SB: Setback; and PT: Pasting Temperature) and (b) process of swelling and rupture of the starch granules during gelatinization and paste obtained.

Briefly, the starch is initially mixed with water, and the suspension is added to the equipment. The mixture is constantly stirred and kept at a temperature (e.g., 50 °C) for a short time (approximately 1 min), being then heated (in general to 95 °C) at a constant heating rate. This stage is when the gelatinization process occurs, which can be seen through the increase in apparent viscosity. The paste is then kept under heating (in general) at 95 °C for almost 3 min, causing the granules to break, with a consequent decrease in apparent viscosity. In this point, the molecules are released, starting to interact among them and with water to form a network. The paste is then cooled (in general to 50 °C) and kept at this temperature for a short time (approximately 2 min), when the molecules start to align (retrogradation), with a consequent increase in the apparent viscosity.

After cooling the paste (at room or refrigerated temperature), the gel is formed. The properties of the starch gel can be determined using a dynamic rheometer, which evaluates the gel continuously at various temperatures, shear rates, and stresses. Some obtained rheological parameters can be useful to evaluate the gels, such as the *G*′ (storage modulus) that represents elasticity; *G*″ (loss modulus) that denotes viscosity; tan δ = *G*″/*G*′ which classifies as solid (tan δ < 1) or fluid (tan δ > 1)).^37,47^

Therefore, it is inferred that rheology is essential to determine important aspects in the development of starch-based biomaterials, such as the development of texture, stability, processability, and functionality of gels. Consequently, understanding the structure and properties of starch is crucial for the biomedical field, allowing for the development of tailored materials with essential properties for different applications.

Furthermore, native starches can be further modified physically, chemically, or enzymatically (for example, by dry heating treatment, ozonation, and enzymatic approaches) to modulate their properties. The starch modification processes result in significant physical and molecular changes that must be considered for starch properties. In fact, many structural changes have been observed in modified starches, for example, partial depolymerization of starch components/reduction in molecular weight, formation of new molecular functional groups (such as carbonyl and carboxyl), changes in granule size distribution and crystalline patterns, as well as formation of porous structure; all those structural changes affect starch properties, such as swelling power, paste and gel properties, gelatinization, and retrogradation.^48,49^ Consequently, the study of starch properties goes beyond the native starches but also involves the various changes that can be promoted by simple or more complex modification processes.

## 3 Potential Starch-Based Biomaterials

### 3.1 Wound Dressing

Skin is the largest organ in the human body and acts as a physical protective barrier to prevent infections and water loss, as well as controlling body temperature.^50^ Skin can suffer damage if exposed to excessive heat, accidents, trauma, and surgery, for example.^51^ The physical damage caused to the skin is a wound, a rupture, or a defect that compromises the structure and physiology of the skin and the body’s processes.^52^

Wounds can be classified according to the number of skin layers affected, as follows:^1^

- Superficial wounds: affect only the epidermis;
- Partial-thickness wounds: extend into the dermis, involving blood vessels, and sweat glands;
- Full-thickness wounds: involve subcutaneous fat and may extend to deeper tissues.

The healing process of a wound is controlled biologically by active substances called growth factors. This tissue regeneration can be divided into two parts: first phase (or lag phase) and second phase. In the lag phase, the hexosamine content increases in the wound fluid, and different polysaccharides and soluble protein precursors of collagen accumulate. The collagen formation begins in the second phase, with an accumulation of cysteine containing proteins, and the concentration of hexosamine falls.^53^

Wound dressings accelerate the healing process by promoting the necessary physiological responses for tissue repair. The composition of these dressings includes analogs of protein and growth factor structures. This must create a necessary environment at the wound site, enabling gaseous exchange and protection from infection.^1^ Moreover, the dressing should be easy to use, comfortable, and conformable to different body parts. Several types of polymeric dressings can be manufactured: films, foams, hydrogels, alginates, and hydrocolloids.^17,52,54,55^ Each of these materials has different properties and applications. The dressings are usually characterized in vitro by their mechanical and absorbent properties, such as tensile strength, strain, and elastic modulus and fluid handling, swelling, water vapor permeability, and gelling, respectively. In vivo tests assess biochemical parameter levels in the granulation tissue of wounds, alongside histological examinations, and mechanical evaluations of the healed tissue.^50^

Starch-based materials have the potential for many biomedical applications, including wound dressings, as they offer advantages in terms of low cost, wide availability, and sustainability when compared to other biopolymers such as cellulose and chitosan. However, as previously mentioned, starch may present some limitations in relation to mechanical properties and high hydrophilic character, which may restrict its application. In addition, unlike chitosan, starch does not exhibit intrinsic antimicrobial activity, requiring modification strategies or the incorporation of bioactive agents to confer antibacterial functionality.^107^ Nevertheless, starch can be chemically or physically modified and combined with other materials to improve usability, ensuring the necessary mechanical and biochemical properties to be useful as a wound dressing.^56^ Next, different studies and methods of combining starch with other materials provide ideal dressings.

Poly­(vinyl alcohol) (PVA) hydrogels are widely used in biomedical applications because of their biocompatibility, film formation, and transparency. This PVA gel shows a high degree of swelling in biological fluid, which simulates natural tissues and is suitable for the body.^50^ Chitosan, a cationic polysaccharide, is used in many applications in tissue engineering, medicine, agriculture, cosmetics, biomaterials, etc. nZnO (nanometric zinc oxide) possesses an antibacterial activity in many cosmetic materials. Hence, the PVA/starch/chitosan (7%) hydrogel improved mechanics and fibroblast viability (>87%); healing rates were 95–96% compared to 79% in the control.^23^

Essential oils extracted from herbs have great antibacterial characteristics; this leads them as potential candidates to use in wound dressings applications. These oils have been used in packaging and in the pharmaceutical industry but rarely incorporated in starch-based membranes. Altaf et al.^55^ proposed incorporating essential oils into PVA/starch-based membranes. They prepared the membranes by a casting method evaluating three different essential oils: oregano oil, tea tree oil, and clove oil. The membranes were prepared with 10% (w/v) of PVA and 7% (w/v) of starch, varying each oil concentration from 0.1 to 0.3 mL. In total, nine samples with three different combinations were tested. The membranes showed great swelling capabilities against water, blood, MgCl_2_ solution, and NaCl solution. This capability evidence the ability to provide a moist environment to the wound area. The mechanical strength decreased by the addition of essential oils, but it was greater than the human skin (>13 MPa). The 0.1 mL clove oil was more effective in improving the mechanical, structural, water sensitivity, and antibacterial properties of membranes, compared to oregano oil and tree tea oil.

In recent years, it has been reported that the strontium ion promotes angiogenesis and prevents bacterial infections. These strontium ion properties lead Mao et al.^17^ to propose a strontium ion cross-linked starch hydrogel (SSH) wound dressing. The SSH hydrogel was prepared with 6% (w/v) strontium nitrate and 2% (w/v) waxy maize starch. The SSH hydrogel promoted the proliferation of fibroblasts, as well a cell viability of 183% in 3 days, and an antibacterial rate above 99.97%. These properties make SSH highly promising for wound dressing applications.

Electrospinning is a method that produces continuous and uniform nanofibers from polymers. Thermoplastic polyurethane (TPU) is a biodegradable polymer that has gained interest in the biomedical sector due to its good biocompatibility and mechanical properties. Mistry et al.^57^ proposed a composite of starch and TPU to produce nanofibers with diameters between 200 and 250 nm. This composite was synthesized with 2.5% (w/v) of potato starch and 7.5% (w/v) of TPU. The starch-TPU nanofibers were cross-linked with various glutaraldehyde concentrations: 5%, 10%, and 15% (v/v). In vivo and histological evaluations showed that starch-TPU nanofibers promote superior wound healing. In 10 days, the wound healing rate of starch-TPU dressings was 79%, while the control group was 73%.

Curcumin is an antibacterial agent found in turmeric; a spice commonly used in eastern foods. To develop a wound dressing that prevents bacterial infection, Hassan et al.^58^ designed PVA/starch membranes containing turmeric for the first time. The membranes were prepared with 10% (w/v) of PVA and 7% (w/v) of starch with different contents of turmeric, from 0 to 2.5 g. The highest antibacterial activity was seen in the PVA/starch membrane with 0.5 g of turmeric. The value of the tensile strength of this membrane was 12.47 0.77 MPa, which is higher than the tensile strength value of human skin (11.5 MPa). The moisture content capacity of the membrane was considered high (91.96% after 6 h) and adequate to promote wound healing. These properties indicate that membranes with turmeric can be applied as a wound dressing.

Citric acid has significant antibacterial effectiveness and can function as a cross-linking agent and plasticizer. Inspired by that, Das et al.^59^ studied PVA/starch/glycerol/citric acid characteristic for wound dressings applications. The glycerol was used to improve film flexibility. The films were fabricated with 3.75% (w/v) of PVA and starch and 2.8% (w/v) of glycerol and citric acid. The elastic modulus and tensile strength values of the films were 28.47 and 2.54 MPa, respectively. The antibacterial activity was observed for both Gram negatives and positive. Despite the relatively low strength, the intermediate stiffness of these films can allow them to adapt to changes in a wound. Furthermore, the antibacterial activity helps prevent infections, which makes this material interesting for use as a wound dressing.

Chitosan is known to easily form gels and films and also to present good biocompatibility and biodegradability. Furthermore, they exhibit antibacterial activity, accelerate coagulation, and promote wound healing. Mixing chitosan to a starch film can improve the mechanical strength and antimicrobial properties. Wu et al.^54^ investigated the effect of amylose content in starch on the film’s properties. They added chitosan to potato starch, corn starch, and glutinous rice starch to prepare the films. The starch-chitosan composite films were prepared with 1% (w/v) starch solution, 2% (w/v) chitosan solution, and 15% (w/v) of glycerol. The amylose content in the films affected their properties. The glutinous rice starch-chitosan composite film revealed the highest tensile strength, elongation, and biocompatibility. These films also were more capable of alleviating inflammation than the other films.

Starch has contributed significantly to the development of wound dressings due to its versatile properties (biocompatibility, absorption/gelation, adhesion, and modifiability) and the potential for modification to overcome its limitations. Its combination with other components, such as PVA, chitosan, and essential oils, as discussed above, and many other materials (Table 1), results in biomaterials with enhanced and additional properties that improve healing (e.g., antimicrobial activity, moisture retention, and drug release). These combinations allow starch-based wound dressings to overcome mechanical and hydrophilic limitations, making them multifunctional and suitable for a wide range of biomedical applications.

**Table 1:** Recent Works Involving the Development of Wound Dressing Based on Starch

biomaterial composition	wound dressing type	production method	findings	reference
blend of starch, chitosan, and poly(vinyl alcohol)	nanofibrous mats	electrospinning technique	the nanofibrous mats showed adequate physical and mechanical properties to wound dressing application, with cytocompatibility, efficiency against Gram-negative and Gram-positive bacteria, and improved wound healing in vitro	Adeli et al.^60^
blend of starch, poly(vinyl alcohol), and glutaraldehyde with graphitic carbon nitride and Silver-deposited Titania nanoparticles	membranes	solvent casting method	the membranes demonstrated excellent breathability (moisture retention of up to 90% oxygen and porosity); slow and sustained release of the drug and complete cure third degree wound in 7 days in an albino rat	Ahmed et al.^50^
blend of starch, poly(vinyl alcohol), and poly(acrylic acid) with pomegranate peel extract	films	solvent casting method	films showed nonhemolytic and antimicrobial activities against Staphylococcus epidermidis and Staphylococcus aureus, noncytotoxicity, and efficiency in almost completely closing scratches in 48 h, tested in vitro	Costa et al.^52^
thiolated wheat starch nanoparticles and porcine skin gelatin	nanocomposite sponges	freeze-drying	sponges with porous and three-dimensional structure, and interconnected pores, acting as NO releasers, collagen secretors, water absorbers and antimicrobials against Escherichia coli; biocompatible and noncytotoxic	Davari et al.^61^
blend of arrowroot starch and gellan gum	scaffold	3D printing	scaffold with desirable structural, physical, and mechanical properties, and ability to host L929 cells with minimal cytotoxicity; potential application as a cell-supporting scaffold to treat skin wounds	Joseph et al.^4^
blend of tapioca starch and poly(vinyl alcohol) with α-terpineol	sheets	electron beam cross-linking	sheets do not demonstrate an inflammatory response in normal rat skin in vivo, improving wound contraction and skin regeneration, with potential application in acid burn wounds	Khalid et al.^62^
blend of starch and gelatin with sorbitol	films	solvent casting method	Films with desirable mechanical, thermal and swelling properties for wound healing, cytocompatibility with human fibroblasts, and good adhesion to the skin	Kozłowska et al.^3^
potato starch with porphyrins	films	solvent casting method	doping starch-porphyrins films photoinactivated Escherichia coli bacterium in a fast way, and favored the in vitro skin wound healing without requiring light	Lopes et al.^63^
corn starch with hydroxyapatite	membranes	solvent casting method	membranes with hydroxyapatite are more resistant, less flexible, and noncytotoxic to osteoblasts. Good adhesion of the osteoblasts and better fracture healing efficiency in vitro	Lucas et al.^56^
blend of corn starch and poly(vinyl alcohol) with zeolite and cellulose nanocrystals	films	solvent casting method	films with a suitable composition for application as a wound dressing, with resistance to deformation like human skin; increasing the corn starch concentration improved the mechanical properties of the films	Threepopnatkul et al.^64^
blend of starch and poly(vinyl alcohol) with AgO and CuO	membranes	solvent casting method	membranes with excellent in vitro physical, mechanical, and antimicrobial properties, and skin biocompatibility, as required to wound dressing application	Uzair et al.^51^

### 3.2 Scaffolds

Bone tissue is a complex connective tissue that makes up most of the human body and performs structural and support functions for other organs. It has regenerative capacity due to the activity of osteogenic cells (osteoblasts, osteoclasts, and osteocytes).^65^ The osteogenic activity of bone tissue can, however, be extensively compromised by injuries caused by trauma, disease, tumors, and infections, for example. The use of temporary extracellular matrices, such as scaffolds, is an excellent alternative to promote osteogenesis and bone repair.^66^ Scaffolds are three-dimensional, porous structures that provide favorable conditions for cell adhesion and proliferation^65^ and can be produced based on polymeric and/or biopolymeric materials. When compared to other polysaccharide-based scaffolds, starch-based scaffolds offer advantages related to their tunable degradation rate and ease of processing, although additional reinforcement or modification is often required to achieve mechanical properties suitable for load-bearing applications.

Starch has been explored in the production of scaffolds for bone tissue engineering for presenting the appropriate criteria such as biocompatibility and the ability to regulate degradation time.^14^ The production methods of scaffolds are varied; among them, one can highlight some more traditional methods such as the casting method by solvent evaporation, fiber bonding, solvent casting and particle leaching, membrane lamination, fusion molding, and more modern methods such as additive manufacturing, where we can cite 3D printing as an example.

In this review, some academic works will be cited that use starch, in its native or modified structure, together with other types of compounds. In addition, different methods of production of these biomaterials will be shown, highlighting the influence of starch on the mechanical, physical, and biological properties of synthesized materials.

Shahriarpanah et al.^67^ developed bioactive carboxylated starch-chitosan composite scaffolds for bone regeneration. The chitosan structure was cross-linked with 2% glutaraldehyde solution. The same solution with pH = 2 was used to cross-link the starch-containing supports (casting method), thus forming the scaffolds. Higher starch content increased the mineralization, strength, carboxyls, and swelling.

Starch-nGO (50%) maximized hydroxyapatite through carboxylic nucleation.^68^ The nanometer graphene was covalently bonded to starch to prepare functionalized starch (SNGO) via an esterification reaction. The starch-based scaffolds were molded in glass vials and then freeze-dried. The SNGO structure containing 50% covalently bound SNGO induces the largest amount of hydroxyapatite crystals. This occurs because the carboxylic groups of nGO behave like poles that attract calcium and phosphate to the surface of the scaffolds, favoring the nucleation of CaP (calcium phosphate).

Goimil et al.^69^ developed poly­(e-caprolactone) (PCL) scaffolds with starch aerogel microspheres and ketoprofen, a nonsteroidal anti-inflammatory drug using supercritical technologies (supercritical CO_2_ impregnation/deposition and foaming). The scaffolds were prepared in varying compositions of starch, ketoprofen, and PCL. These compounds were mixed and placed in cylindrical molds and were inserted into a high-pressure stainless-steel autoclave. After this step, depressurization was performed, and then the scaffolds were stored overnight to allow complete desorption of CO_2_. The scaffolds containing starch aerogels showed an increase of porosity and pore interconnectivity to promote bone tissue growth processes at the expense of a small decrease in mechanical properties.

Mirab, Eslamian, and Bagheri^22^ developed a highly porous starch/poly­(vinyl alcohol) (PVA)-based nanocomposite scaffold incorporating different bioadditives including citric acid, cellulose nanofibers, and hydroxyapatite (HA) nanoparticles. The scaffold was prepared by employing one-way and cryogenic methods, such as freeze casting, followed by freeze-drying. Based on the mechanical test results, the cellulose-HA-reinforced starch-based scaffold possesses sufficient compressive modulus and yield strength for no-load applications in the dry state, and it also exhibits rapid shape recovery in the wet state.

Koski et al.^70^ aimed in their studies to understand the use of a biodegradable polymer binder system (PCL) in starch-based composite scaffolds using a ceramic paste-based free form solid fabricator (SFF). The scaffolds were produced by 3D printing of the extrusion type, and with the analyses of the results, it was shown that the starch loading would improve the mechanical strength from 4.07 to 10.35 MPa, which left the material with the mechanical strength close to cancellous bone. In addition, it was found that the use of starch as a natural binder system in the scaffolds improved both compressive strength and biocompatibility in vitro.

Koski and Bose^14^ evaluated the effect of amylose content on the mechanical and physical properties of starch-HA composite scaffolds for bone and tissue engineering applications. Starch-HA composite scaffolds using corn, potato, and cassava sources of gelatinized starch were fabricated by using a self-designed solid free-form fabricator (SFF) and were constructed by 3D printing. Based on the results, the mechanical strength of the starch-HA scaffolds would increase with increasing amylose content based on the botanical source (corn, potato, and cassava) and the added weight percentage.

Gutiérrez-Sánchez et al.^27^ had as objective in their work the in vitro evaluation of supports obtained from mixtures of starch with poly­(lactic acid) (PLA), treated with peptides of arginine-glycine-aspartic acid (RGD). The scaffold was obtained by using the electrospinning technique. Proliferation assays show that osteoblasts proliferate better on PLA and PLA surfaces with 5.0% starch scaffolding. Furthermore, it was verified in this study that the morphology of the polymeric support is modified by the presence of starch, and as the amount of starch increases, more irregular surfaces are obtained under the same operating conditions, concluding that in this case, the surface modification does not alter the properties of the biomaterial.

Gunawan et al.^71^ developed a scaffold based on sweet potato starch and hydroxyapatite. The scaffolds were fabricated by the sintering process, which was evaluated at different temperatures. It was observed that the higher the starch content in the product, the more porous the scaffold became at a temperature of 1050 °C, and furthermore, there was better compressive strength at a temperature of 1200 °C.

Chhabra et al.^72^ in their studies developed a scaffold by combining the properties of two dressing biopolymers: starch and gelatin, enhancing their mechanical strength by cross-linking. The scaffolds were manufactured by a freeze casting process where the biopolymer mixture was gently poured into 24-well plates and kept for freezing (−40 °C) for 24 h and then freeze-drying. After in vivo and mechanical property studies, these highly porous starch-gelatin based constructs allowed excellent cell infiltration and showed histological similarities to human skin.

Prasopdee et al.^73^ developed 3D scaffolds based on carbohydrates, bovine serum albumin (BSA), and cassava starch. The scaffolds were produced by the freeze casting process followed by freeze-drying. Using this method, BSA/starch generated a stable foamlike scaffold, while BSA alone failed to maintain a rigid structure. In addition, the albumin/starch scaffolds were found to possess a porous structure. It is concluded then that the presence of starch increases the mechanical strength and porosity.

The main findings of the studies discussed above on starch-based bone scaffolds highlight their strong potential for bone tissue engineering, particularly for bone replacement applications. Additional examples and their key outcomes are summarized in Table 2. Overall, starch has contributed to the development of functional bone scaffolds by enhancing properties such as adhesion, swelling behavior, biodegradation rate, cell proliferation, and mineralization, which are essential for osteogenesis. Moreover, the versatility of starch to incorporate different components and form polymer blends enables further optimization of the mechanical and physical properties of these scaffolds. Where available, quantitative mechanical data (e.g., tensile strength, compressive strength, and elastic modulus) are reported, with values typically ranging from approximately 4 to over 10 MPa, depending on the composition and processing route.

**Table 2:** Recent Works Involving Development of Scaffolds Based on Starch Have the Potential to be Applied in Bone Tissue Engineering

biomaterial composition	production method	findings	reference
starch cross-linked with siloxane	freeze-drying	scaffolds with hydrophilic and microstructural properties suitable for bone regeneration applications. Good adherence of bone marrow mesenchyme stem cells, without cytotoxicity or cell death. There was mineralization of the scaffolds after immersion in simulated body fluid solution, confirming their potential use	Aidun et al.^74^
blend of corn starch and poly(3-hydroxybutyrate)	electrospinning technique	the diameters of the scaffold fibers decreased with the addition of starch, while the thermal stability, mechanical properties, hydrophilicity, and degradation rate increased. Furthermore, the addition of starch improved the cell viability and calcium mineralization of the scaffolds	Asl et al.^75^
blend of potato starch and polycaprolactone with CaO nanoparticles	electrospinning technique	scaffolds with good distribution of CaO particles facilitated by the presence of starch, increased Young’s modulus, high biomineralization potential (hydroxyapatite formation after 28 days), noncytotoxicity in vitro supporting cell adhesion and viability, and good degradation and reabsorption in vivo, with potential application in bone regeneration	García et al.^66^
blend of corn starch and alginate or chitosan with nanohydroxyapatite and *S*-nitroso-*N*-acetylpenicillamine	freeze-drying	scaffolds with NO release eradicated >99% of Gram-positive and Gram-negative bacterial strains, exhibits excellent compression resistance, and are noncytotoxic to mouse fibroblast cells. Potential application in bone regeneration, for the growth of osteoblasts, angiogenesis, and regulation of gene and tissue expression at the site of bone injuries and infections	Pant et al.^76^
starch granules with hydroxyapatite	solvent casting method	scaffolds with micrometer porous and dense structure suitable for application in bone regeneration, in addition to being noncytotoxic to the mammalian cell line	Ragunathan et al.^77^
starch with bioactive glass, quail eggshell, and Aloe vera	freeze-drying	scaffolds with an interconnected porous structure, which can provide cell adhesion and proliferation, increased the expression of osteocalcin and osteopontin bone proteins and the osteogenic response of MG-63 cells. They were biocompatible and supported the formation of calcium deposits, demonstrating potential application in bone regeneration	Soltani and Alizadeh^78^
potato starch modified by dry heating treatment	3D printing	the modified starch formed stronger hydrogels, depending on the formulation, which presented greater printability, resolution, and fidelity of the scaffolds. The scaffolds showed better mechanical properties, lower biodegradability rate, and swelling power, with potential application for bone regeneration	Sponchiado et al.^79^
corn starch with nanohydroxyapatite	solvent casting method/and salt-leaching	scaffolds with higher starch concentration showed high dielectric polarization and loss upon exposure to applied electric field, stronger intermolecular interaction with nanohydroxyapatite, porosity, effective surface areas, and higher biodegradation rate. Scaffolds exhibit potential application for bone regeneration	You et al.^80^

### 3.3 Drug Delivery Systems

In addition to various applications of starch as a raw material to produce bone scaffolds and wearable devices, for example, this polysaccharide has also been explored as a biomaterial to function as a sustained release agent. Drug delivery systems (DDS) are formulations or devices developed to improve the pharmacokinetics of a drug by controlling its release at the desired site of action.^81^ Besides sustained release, in some cases, DDS can also provide protection to the carried drug, increasing its safety and bioavailability when released in the targeted location. The composition of the DDS can vary from proteins and inorganic materials to synthetic and biopolymers, such as polysaccharides.^82^

Usually, micro- and nanocarriers liberate the therapeutic agent in response to the local variation of specific physicochemical parameters of the affected region, such as temperature, pH, surface area, pressure, and solubility.^83^ The intrinsic biodegradability of polysaccharides, like starch, presents an additional advantage for the controlled release of the drug, reducing the undesirable side effects of burst liberation.^84^ Figure 4 illustrates the starch-rich sources to produce different types of DDS.

**Figure 4:**
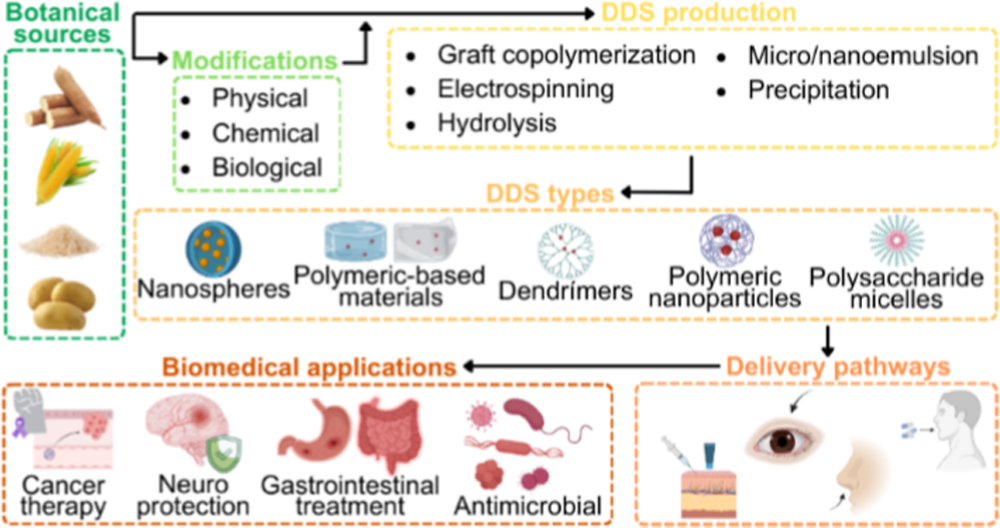
Schematic representation of different sources of starch, routes of starch modification, and types of drug delivery systems (DDS), along with their major biomedical applications.

Recently, various polysaccharides have been explored as a base material for DDS, as they naturally exhibit interesting characteristics for these systems, such as biodegradability, nontoxicity, low-cost acquisition on a large scale, and the possibility of being chemically modified.^85^ In general, the carriers can be presented as different types of materials and sizes, ranging from macro to nano, such as hydrogels, liposomes, micelles, quantum dots, polymeric systems, and nanomaterials (nanoparticles, nanocapsules, and nanospheres).^81,86,87^ However, for polysaccharide-based DDS, some authors^82,88^ have divided their design into three major groups: (a) polysaccharide–drug conjugated carriers, which are devices formed by the attachment of polysaccharides and drugs through noncovalent interactions or covalent bonds, using chemical reactions such as oxidation, esterification, or etherification, for example;^89^ (b) polysaccharide-based particles made by a self-assembly process with drug loading, such as micelles and liposomes for hydrophobic and hydrophilic drugs, respectively;^90^ and (c) polysaccharide-based hydrogels with encapsulated therapeutic molecules, mainly through noncovalent interactions.^91^ Table 3 shows some studies that have developed different starch-based biomaterials with therapeutic molecules for biomedical applications.

**Table 3:** Recent Works Involving the Development of Starch-Based Biomaterials with Therapeutic Molecules for Biomedical Applications

starch origin	biomaterial composition	therapeutic molecule loaded	production method	route of administration/application	findings	reference
wheat	dialdehyde starch (DAS)-chitosan hybrid hydrogels	betamethasone	gelation method at room temperature	ophthalmic drug therapy	high content of DAS in hydrogels improved gelation, resistance, degradation, and in vivo and in vitro release rate; biocompatibility with in vitro human umbilical vein endothelial cells	Aslzad et al.^94^
corn with high amylose content and potato (low amylose)	corn and potato starch nanoparticles (CSN and PSN)	vitamin D_3_	ultrasonication	biomedical applications	NPs obtained with an average size <100 nm; vitamin D_3_ was successfully entrapped in nanoparticles and presented a higher thermal stability due to the NPs protection	Hasanvand et al.^95^
normal maize (NM, low-amylose) and Hylon VII (H7, high-amylose)	starch-based hydrogels	vanillin (VNL), 5-fluorouracil (5-FU), and doxorubicin (DOX)	high temperature–pressure gelatinization	orally administrable for endemic colorectal pathologies	prolonged drug release in the large intestine (15–56% after 24 h)	Koev et al.^96^
waxy maize	starch microparticles (SMPs)	curcumin-cyclodextrin complex (CUR-CD@SMPs)	self-assembly of the short chain glucans	oral route for gastrointestinal diseases	improvement in the stability of curcumin after encapsulation; development of size-controlled SMPs; the increase in the size of microparticles influenced proportionally the thermodynamic stability of SMPs and affected inversely the release rate of CUR-CD	Luo et al.^97^
corn	semi-IPN hydrogels of carboxymethyl starch (CMS) and poly(2-dimethylaminoethyl methacrylate, PDMAEMA)	ibuprofen (IBU)	radical polymerization	biomedical applications	improvement of cytocompatibility (fibroblast cells), pH, temperature sensitivity, and rheological behavior with the addition of CMS in the hydrogels composition	Nita et al.^98^
normal maize	modified starch nanovesicles (amylolipids, ALNs)	curcumin	microemulsification	intranasal route for treatment of brain injuries	ALNs with increased stability and brain delivery; ALNs enhanced brain bioavailability of curcumin	Sintov^99^
corn	starch-modified alginate nanoparticles	theophylline (THP) and bovine serum albumin (BSA)	ionotropic gelation technique	oral drug delivery for biomedical applications	drug release with a pH-dependence (released in intestinal fluid); NPs with zero toxicity and biocompatibility	Thomas et al.^100^
rice	acetylated starch nanocrystals (ASN)	doxorubicin hydrochloride (DOX)	ASN prepared by acid hydrolysis (sulfuric acid)	cancer therapy	biocompatibility; ASN with higher drug loading content and loading efficiency than the native starch; decreased IC_50_ values of DOX-loaded ASN to HeLa cells	Xiao et al.^101^
not described	cholesterol/imidazole-modified oxidized-starch nanoparticles (Cho-Imi-OS)	curcumin (Cur-NPs)	self-assembly through dialysis	cancer therapy	pH-triggered drug release property; increased cytotoxicity of Cur-NPs on A549 cells (human lung cancer cell line)	Xu et al.^102^

To obtain these materials at both nano- and microscales, various methods have been employed, such as micro- and nanoemulsion, ultrasonication, electrospinning, and other chemical techniques like hydrolysis and graft copolymerization.

Microemulsion is widely used for synthesizing nanoparticles due to several advantages it offers over nanoparticles, such as controlled size, homogeneity, and thermodynamic stability.^92^ This technique can be classified based on dispersion properties: as water-in-oil (W/O) emulsion, where the hydrophilic domain is dispersed in the hydrophobic phase (oil phase), stabilized by surfactant molecules, and as oil-in-water (O/W) emulsions, where the process occurs in reverse to the W/O method described.^83^ On the other hand, nanoprecipitation is based on the successive addition of a solvent (first system) into a nonsolvent (second system) that are miscible in each other, with the polymer and drugs typically dissolved in this first system.^93^

Through the W/O microemulsion method, Xie et al.^103^ produced acid-treated cassava starch nanoparticles loaded with paclitaxel, a medicine used to treat cancer. The nanoparticles were obtained with an average size of 252 nm and a maximum loading rate of 36.14 mg/g. At pH 7.0 and 5.0, the release rate of paclitaxel was 37.61% and 58.65% in 96 h, respectively. The higher release rate at pH 5.0 can be attributed to the hydrolysis of phospholipid cross-links under acidic conditions. The nanoparticles produced in this study could be applied as biocompatible and biodegradable release controls of drugs such as paclitaxel.

As a top-down approach, ultrasonication is an eco-friendly method used to produce micro and nanoscale DDS. This technique is based on the utilization of ultrasonic waves (with a frequency >20 kHz) to induce the fragmentation of the material. To produce starch-based DDS, ultrasonication can reduce the length of amylopectin and amylose by breaking their covalent bonds within the biopolymer due to the intense mechanical effects caused by the collapse of bubbles generated by ultrasonic waves.^83^ Ahmad and Gani^104^ developed starch-based nanoparticles by sonication (10 min at 60 kHz) with starches from different botanical sources (horse chestnut—HS, water chestnut—WS, and lotus stem starch—LS) and resveratrol, a bioactive compound with anti-inflammatory, neuroprotective, and antidiabetic activities that can be found in some plant-based foods. The nanoparticles were obtained with a hydrodynamic particle size ranging from 400 to 700 nm and high negative zeta potential values, indicating the great stability of the NPs. The encapsulation efficiency (EE) of the three starch NPs was found to be around 75–81%. According to the authors, this demonstrates the ability of starch NPs to entrap the resveratrol in their core, especially for HSR NPs, which exhibited the highest EE % (81.46%) and a porous morphological structure that facilitated the encapsulation of the therapeutic agent in its core.

In addition to the methods described above, the hydrolysis of starch granules has been used to produce certain DDS, utilizing both chemical (acid or alkali) and enzymatic hydrolysis. Starch hydrolysis can occur in two stages with different reaction rates. Initially, the hydrolytic reaction targets the amorphous region of starch granules, where it proceeds more rapidly. It subsequently advances to the more crystalline region, where the reaction continues at a slower rate.^105^ Xiao et al.^101^ produced acetylated starch nanocrystals with encapsulated doxorubicin (DOX-ASN), using broken rice. By utilizing the acid hydrolysis method, the ASN were obtained with sizes between 80 and 700 nm, depending on the degree of substitution of acetylated starch (AS). Moreover, when compared to the AS control (at macro scale), doxorubicin was encapsulated more efficiently within the nanocrystals (ASN), with an encapsulation efficiency of approximately 80–90%. In comparison to free DOX, the nanocrystals were also able to enhance its effectiveness against HeLa cells, reaching 70% of cell mortality (while for free DOX, only 50% cell mortality was achieved) through controlled release over a period of up to 10 h. The results obtained demonstrate the efficiency of starch nanocrystals obtained by acid hydrolysis as a drug delivery system.

Finally, the studies shown above demonstrate the versatility of starch-based biomaterials to be used in various biomedical applications as efficient drug delivery systems. As a biopolymer, starch offers a safe and biodegradable system for drug encapsulation. Its porous structure allows for the efficient incorporation of drugs, while its ability to form gels and micro- and nanocomplexes enables control of drug release over time, protection, and increased bioavailability. Compared with other commonly used polysaccharide-based drug delivery systems, such as cellulose and chitosan, starch-based carriers exhibit greater versatility in chemical modification and structural design, enabling precise tuning of release profiles and interactions with different classes of therapeutic agents. In this context, the chemical modification of starch plays a key role in adjusting drug compatibility and achieving controlled release behavior.

Although a wide range of starch-based drug delivery systems has been reported, comparative analysis reveals clear trends in release kinetics and biopharmaceutical performance. Systems based on starch nanoparticles and nanocrystals generally exhibit sustained release profiles, which are advantageous for improving the drug stability and prolonging the therapeutic action. In contrast, chemically modified starch systems and hybrid formulations often display pH-responsive or stimulus-triggered release, enabling site-specific drug delivery, particularly for intestinal and cancer-related applications. These differences in release behavior directly influence biopharmaceutical performance, as controlled and responsive release profiles are associated with enhanced bioavailability, reduced burst release, and improved therapeutic efficacy. However, variability in experimental conditions and the lack of standardized release protocols limit direct quantitative comparisons across studies.

Despite the growing number of starch-based drug delivery systems reported, a vast majority of studies remain restricted to in vitro or early preclinical evaluation, with only a limited number progressing to in vivo validation and virtually no clinical translation. To bridge this gap, future research should prioritize pH-responsive and stimulus-triggered platforms and follow a clear translational roadmap, including standardized in vitro testing under physiologically relevant conditions, short- and long-term in vivo studies addressing biocompatibility, degradation, and immune response, as well as early consideration of regulatory and scalable manufacturing constraints to enable site-specific delivery and improved therapeutic performance.

### 3.4 Hybrid and Smart Starch-Based Biomaterials

Recent advances have increasingly explored hybrid starch-based biomaterials that integrate nanomaterials or smart polymers to impart stimuli-responsive behavior. Starch, as a biocompatible and biodegradable matrix, can be chemically modified or combined with other polymers to enhance functionality beyond passive structural support.^108,109^ For instance, incorporating inorganic nanofillers such as Fe_3_O_4_ nanoparticles or other inorganic particles into starch hydrogels has been shown to enhance properties relevant for controlled drug release and biomedical uses.^110,111^ Magnetic starch-based nanohydrogels exhibited stimulus-responsive release profiles in drug delivery systems, highlighting the potential for multifunctional therapeutic platforms.^110^

Recent work has also focused on starch nanocomposite hydrogels, where interactions between nanoparticles and polymer chains influence dispersion and structural stability, which are critical for mechanical reinforcement and functional performance in biomedical applications.^112^ Moreover, strategies to design hybrid systems that combine starch with synthetic polymers such as polyacrylamide and nanomaterials like graphene quantum dots (GQDs) have demonstrated targeted delivery capabilities in advanced therapeutic contexts, such as delivery across biological barriers (e.g., blood–brain barrier).^113^

In parallel, the development of stimuli-responsive starch systems has leveraged environmental triggers such as pH and enzyme activity for adaptive release kinetics. pH-sensitive starch-based hydrogels have been shown to exhibit controlled swelling–deswelling behavior influenced by the surrounding medium, enabling more precise drug release profiles.^108^ The inherent hydrophilicity and tunable architecture of starch hydrogels support their use as drug carriers and scaffolds that can facilitate controlled drug delivery and tissue regeneration, although challenges remain in optimizing long-term stability, reproducibility, and scalable manufacturing.^108,109,112^ Systematic evaluations of in vivo performance and standardized protocols are still limited, indicating clear directions for future research to bridge laboratory studies with clinical translation.

## 4 Research Gaps and Perspectives

Despite the significant progress achieved in the development of starch-based biomaterials, several critical limitations still hinder their translation from laboratory-scale studies to clinical and industrial applications. One of the most persistent challenges is mechanical weakness, particularly for wound dressings and load-bearing scaffolds, often necessitating polymer blending, cross-linking, or reinforcement with inorganic fillers to achieve application-relevant performance. In parallel, the literature reveals a pronounced lack of standardized quantitative data on mechanical properties and biodegradation time, which limits predictive material design and hampers direct comparison across studies.

In this review, notable achievements in starch-based biomaterials developed as wound dressings, scaffolds, and drug delivery systems are summarized, highlighting their potential to support tissue regeneration and enable the localized delivery of regulatory or therapeutic agents. These systems can be tailored through different combinations of starch with complementary polymers, allowing for modulation of biochemical and functional properties to meet specific regenerative demands. However, the absence of harmonized characterization protocols constrains the rational selection and optimization of these systems for translational use.

Beyond material performance, several technological challenges remain central to clinical translation. Scalability represents a major bottleneck, as most fabrication strategies rely on laboratory-scale processes with limited evaluation of reproducibility, cost efficiency, and continuous manufacturing potential. Sterilization also remains insufficiently addressed, despite its critical importance for biomedical applications since conventional sterilization methods may alter material structure, mechanical integrity, or degradation behavior. Additional translational barriers include storage instability of biomaterials incorporating cells or growth factors and the difficulty in achieving long-term controlled release capable of effectively modulating the local microenvironment, promoting functional recovery, or providing sustained trophic and anti-inflammatory effects.

Based on this analysis, the main research gaps, closely linked to specific technological challenges, can be prioritized as follows:

- Standardization of biodegradability and mechanical testing, including evaluation under physiologically relevant conditions, to enable cross-study comparability and predictive material design.
- Optimization of structural, biomechanical, surface, and degradation properties to improve cell–material interactions and extracellular matrix deposition according to the intended application.
- Integration of multiple biomaterials and bioactive molecules with advanced manufacturing approaches (e.g., bioprinting and microfabrication) to achieve spatial control, avoid burst release, and prevent premature inactivation.
- Promotion of neurovascularization and development of multitissue scaffolds to reduce localized necrosis, enhance implant integration, and support functional tissue regeneration.
- Simplification of manufacturing workflows and transition toward scalable, reproducible, and cost-effective production routes, enabling mass production beyond laboratory-scale environments.

Addressing these challenges is essential to advance starch-based biomaterials from preclinical screening to reliable clinical applications. From a translational perspective, systematic assessment of economic feasibility, process scalability, and unit operations, such as drying and solvent exchange, will be critical, as most current studies rely on empirically optimized protocols. The development of continuous processing models may further improve the process understanding and industrial viability.

Although significant progress has been made in characterizing morphological and structural features, the lack of standardized testing protocols continues to limit the data comparability. In particular, quantitative information about biodegradation time and mechanical performance under relevant physiological conditions remains scarce, despite frequent qualitative claims of biodegradability and adequate mechanical behavior. Finally, ongoing research into biomaterial–immune system interactions is expected to provide valuable insights into immunomodulatory design principles, guiding the development of advanced starch-based biomaterials that actively promote implant integration and functional tissue regeneration.^106^

## 5 Conclusion

Due to their biocompatibility and similarity to extracellular matrices, starch-based biopolymers have been widely explored for wound dressings, scaffolds, and drug delivery systems; however, limited mechanical and chemical stability under physiological conditions still poses fabrication challenges. To overcome these limitations, biopolymers are commonly chemically modified or blended with other polymers with the chosen strategy depending on the biopolymer chemistry, intended application, and target physiological environment. The choice of the preparation method hinges on the chemical nature of the biopolymer, the specific biomedical application, and the anticipated physiological conditions to which the biomaterial will be exposed.

Because biomaterials are susceptible to microbial contamination, appropriate sterilization strategies are essential prior to their clinical use. The growing demand for tissue and organ substitutes continues to drive the development of advanced starch-based biomaterials with increasing emphasis on immunomodulatory design, translational validation, and scalable manufacturing strategies. Additionally, the future research agenda includes the development of biomaterials for early-stage disease diagnosis and treatment.
